# The impact of perennial allergic rhinitis with/without allergic asthma on sleep, work and activity level

**DOI:** 10.1186/s13223-019-0391-9

**Published:** 2019-12-06

**Authors:** Mercedes Rodriguez Romano, Stephanie James, Emily Farrington, Richard Perry, Lisa Elliott

**Affiliations:** 1 ALK, Bøge Alle 1, 2970 Hørsholm, Denmark; 2 Adelphi Values, Macclesfield, UK

**Keywords:** Allergen immunotherapy, House dust mite, Sleep disorders, Burden of disease, Quality of life, Real-life study

## Abstract

**Background:**

Allergic respiratory diseases such as allergic rhinitis (AR) and allergic asthma (AA) are common conditions that can influence sleep and daytime functioning. However, the significance of this impact is unclear—particularly in perennial allergy sufferers. This study investigates the impact of perennial allergy on sleep, daily activities and productivity.

**Methods:**

Adults with self-reported or physician-diagnosed perennial AR aged ≥ 18 years were recruited in Denmark, France, Germany and Sweden. Allergy sufferers were identified using online panels closely matching national population characteristics for each country. Impact on sleep, work, productivity and activity (by the Work, Productivity and Activity Index) were analysed. Descriptive analyses were conducted.

**Results:**

In total, 511 subjects with perennial AR (47.4% also with seasonal allergy) completed the survey. Most subjects (77.5%) had a physician-diagnosis of AR; 46.4% were diagnosed with both AA and AR. Most subjects (65.2%) reported sensitisation to house dust mites. Of all subjects, 66.0% reported sleep problems. Subjects with sleep problems woke, on average, 3.8 times per night, with 92.0% taking 15+ min to fall asleep (22.2% took 60+ min). Upon waking at night, 40.8% struggled to get back to sleep, and 69.2% had difficulties waking in the morning due to tiredness. Disturbances in daily functioning due to sleep issues were reported in 85.5–95.0% of subjects with sleep problems across all aspects investigated. Overall work and activity impairment were 53.3% and 47.1%, respectively. Sleep issues were more frequent (78.1% vs 54.7%) in those diagnosed with both AR and AA compared to AR alone, and more burdensome, with a greater impact on daily functioning (47.0% vs 33.3%) and impairment in work and activity (62.0% and 54.9% vs 39.3% and 35.2%, respectively). Of all subjects, 20.5% were receiving AIT at the time of the survey; of these, 36.4% reported moderate or great improvement in sleep due to allergy treatment.

**Conclusions:**

In perennial AR sufferers, sleep problems are common and impact on daily functioning, with results indicating a greater burden in those with both AR and AA compared to AR alone.

## Introduction

Allergic rhinitis (AR) occurs when airborne allergens trigger an allergen-specific immunoglobulin E mediated inflammatory response, often resulting in nasal obstruction, sneezing, itching or rhinorrhea [1]. AR is extremely common, particularly in Western countries. The World Health Organisation (WHO) estimates that approximately 400 million people suffer from AR worldwide [2] and previous studies have estimated the prevalence in France at 25.4%, in Germany 20.6%, in Sweden 30.9% and in Denmark 23.1% [3–5]. AR is often associated with allergic asthma (AA): approximately 25% of people with AR also have asthma and approximately 50% of people with asthma have AR [6–9].

AR can be triggered by perennial or seasonal allergens or a combination of both [10]. House dust mites (HDMs) are the primary cause of perennial respiratory allergies [10]. As HDMs are difficult to eliminate in the sleep environment, they can negatively affect sleep. For example, a cross-sectional study (N = 1786) indicated a causal link between HDM AR and sleep problems, with the majority of subjects (74%) reporting their sleep problems had prompted them to consult their physician [11]. Furthermore, in both a prospective observational Spanish study (N = 2275), and a controlled cross-sectional French study (N = 591) a positive correlation was seen between sleep disorders and AR [12, 13].

The current standard of care for perennial AR focuses on pharmacotherapy, such as taking antihistamines daily to provide temporary relief of symptoms and allergen avoidance, by reducing indoor exposure—for example encasing bedding in allergen-impermeable fabric [1]. If people still have inadequately controlled moderate-to-severe AR following these measures, allergen immunotherapy (AIT) may be offered by their physician; however, in practice many wait years with inadequately controlled AR before they are offered AIT. AIT is the only treatment with the potential to alter the course of disease, having demonstrated multiple clinical benefits in perennial AR sufferers including sustained symptom control and reduction in medication use [1, 14–18]. Likewise, quality of life improvements following AIT have been documented and clinical results indicate that AIT has the potential to improve AR-related sleep issues [19].

Previous studies have established a link between AR and sleep [13, 20]. However, the frequency/severity of the sleep problems and their subsequent impact on quality of life remains uncertain—especially in perennial allergy sufferers with additional allergic asthma. The objectives of this study were, therefore, to understand the characteristics, management and burden of perennial allergy (AR with or without AA) in terms of sleep disturbance and impact on daily life, work and activity.

## Methodology

### Study design and participants

This was an online market research survey administered to panels of subjects with allergies in Denmark, France, Germany and Sweden, between November 3rd, 2018 and December 1st, 2018. Sampling was geographically representative in terms of reflecting overall population distributions within each country, but not designed to match any geographic/demographic quotas identified in previous AR studies. Participants consent was collected both before inclusion in the online panel and before opting into this survey.

A pilot study tested functionality and routing of the screener, robustness in capturing appropriate respondents and testing of participation levels. After the pilot study, adjustments to the wording and the routing through the survey questions were made to collect the required quotas in the main fieldwork. The quota was 100–200 in each market—set to obtain data that was sufficiently robust for individual country analysis. The incidence of perennial AR in the literature appeared to far exceed that encountered in the pilot study [3–5]. Therefore, full fieldwork required a longer ‘live’ period in order for a higher number of potential participants to be approached than initially anticipated.

### Survey development

A study screener was developed to ensure that those who confirmed that they had perennial AR, by self-report or physician-diagnosis, proceeded to the main survey. Those who had seasonal allergies only (i.e. no perennial allergy) were excluded. The main survey (Additional file 1) included questions regarding: disease characteristics [including determination of severity according to Allergic Rhinitis and its Impact on Asthma (ARIA) criteria, and diagnosis of AA], sleep problems and treatment management. Questions regarding sleep investigated the severity, frequency and impact of sleep problems. Treatment questions were developed to capture over the counter (OTC) and prescription medication use. All sleep and treatment questions were based on a recall period of the past month—long enough to account for weekly variations but short enough to minimise recall bias. The impact of sleep problems on productivity was assessed in employed subjects by the Work Productivity and Activity Index (WPAI)—a six question, validated tool which can be administered online. The WPAI was adapted to assess the impact of sleep as the specific health problem in line with WPAI guidance [21]. The WPAI assesses the following in the past 7 days: hours missed from work due to sleep issues, hours missed from work due to other reasons, hours actually worked, the degree to which sleep issues impair productivity whilst working (0–10 scale; higher score indicate greater impairment) and the degree to which sleep issues affect regular activities. The average work time missed (absenteeism), impairment while working (presenteeism), overall work impairment (absenteeism plus presenteeism) and activity impairment were calculated in line with WPAI guidance, whereby scores are expressed as percentages and higher scores indicate greater impairment [21].

Subjects were routed to specific sets of questions in the sleep and treatment sections of the study (Additional file 2), to ensure that the questionnaire flowed logically. Specifically, subjects who reported sleep problems which were not related to allergy were routed to the general sleep questions, and subjects who reported their sleep problems were related to allergy were routed first to allergy-specific sleep questions prior to the general sleep questions. The full survey was designed to take no more than 15 min for subjects to complete, to maximise completion rates.

### Data analysis

All data were anonymised and descriptive analyses were conducted for the full data set, and sub-analyses were conducted for diagnosis and treatment groups.

For the diagnosis sub-analysis, subjects with a physician diagnosis of AR and no diagnosis of AA (i.e. AR only) versus all subjects with both a physician diagnosis of both AR and of AA were compared. For the treatment sub-analysis, those receiving AIT at the time of the survey versus those who had received other prescribed treatments (but not AIT) were analysed. Those receiving other prescribed medication were deemed the most appropriate comparator to the AIT group, as they were likely to have more severe disease compared to those not receiving prescribed treatment. Routing through the questionnaire is presented in Table 1.

**Table 1:** Routing through questionnaire sections and subgroups used in analyses

	Total N (%)
Population characteristics	511/511 (100%)
Treatment management	511/511 (100%)
Sleep problems (frequency and impact)	338/511 (66.1%)
WPAI	325/511 (63.6%)
Sub-analyses groups	
Physician diagnosis: AR only (no AA)	159/511 (31.1%)
Physician diagnosis: AR and AA (no AA)	237/511 (46.4%)
Treatment: receiving AIT	105/511(20.5%)
Treatment: receiving other prescription medication (not AIT)	110/511(21.5%)

All 511 subjects were routed to all sections of the survey, except for the ‘sleep problems’ to which only those who reported sleep problems (N = 338) were routed. Only those who reported employment were included in the WPAI analysis

## Results

### Population characteristics

A total of 511 subjects with perennial allergies completed the survey: Germany 35.0% (N = 179); France 28.4% (N = 145); Sweden 19.2% (N = 98) and Denmark 17.4% (N = 89). Subject characteristics are shown in Table 2.

**Table 2:** Characteristics of disease in full sample (N = 511)

	Category	Percentage (%)	N
Age	Total	–	511
	18–24	26.2	134
	25–34	21.9	112
	35–44	14.7	75
	45–54	22.1	113
	55–99	15.1	77
Sex	Male	46.0	235
	Female	54.0	276
Smoking status	Yes	52.4	268
	No	47.6	243
Severity	Mild/persistent	1.2	6
	Moderate-severe/persistent	61.3	313
	Mild/intermittent	1.6	8
	Moderate-severe/intermittent	36.0	184
	Denmark	17.4	89
	France	28.4	145
	Germany	35.0	179
	Sweden	19.2	98

The majority were categorised as having moderate-to-severe, persistent disease (61.3%; N = 313) or moderate-to-severe, intermittent disease (36.0%; N = 184), by ARIA criteria. A physician diagnosis of AR alone or AR and AA was reported in 31.1% (N = 159) and 46.4% (N = 237) of subjects, respectively, with the remaining 22.5% (N = 115) having self-reported AR. All subjects had perennial allergies and 47.4% (N = 242) had both perennial and seasonal allergies.

**Fig. 1:**
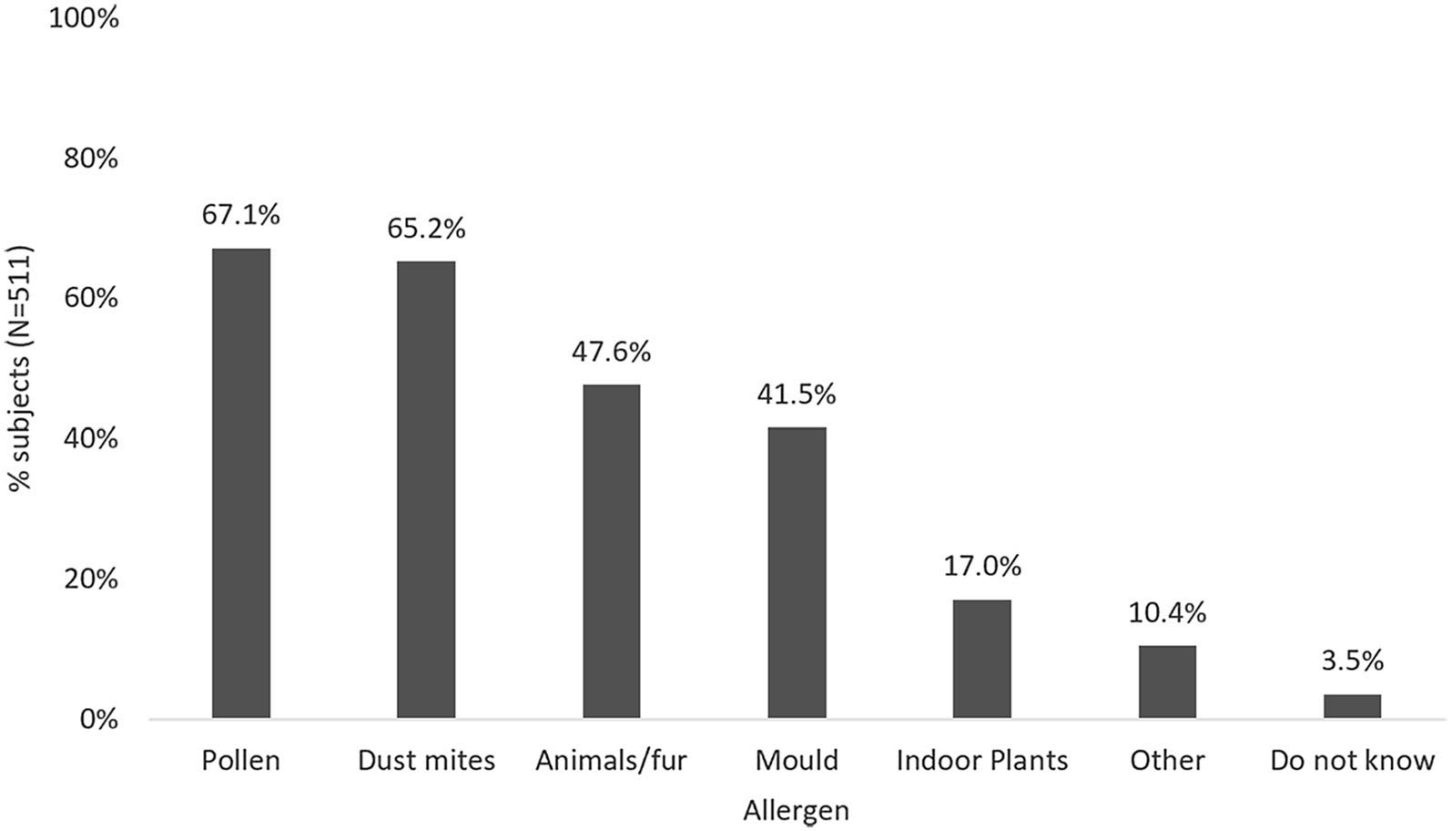
Reported allergy causes in full sample of perennial allergy sufferers

The most commonly reported allergy causes were pollen (67.1%; N = 343), HDMs (65.2%; N = 333), mould (51.6%; N = 212), animals/fur (47.6%; N = 243), and indoor plants (17.0%; N = 87) (Fig. 1).

Polysensitisation to three or more allergens was reported by the largest proportion of subjects (49.3%; N = 252); two were reported by 24.1% (N = 123); one by 23.1% (N = 118) and none reported by 3.5% (N = 18) subjects.

The season in which subjects reported that their worst allergy symptoms were experienced was Spring (48.1%; N = 246), followed by Summer (40.3%; N = 206), Autumn (23.5%; N = 120) and Winter (10.6%; N = 54). Some subjects noted that their worst symptoms occurred in more than one season; however, a proportion of subjects (30.5%; N = 156) reported that their symptoms were the same all year.

### Treatment management

Half of all subjects with perennial allergies (50.3%; N = 257) used OTC medication to help their sleep. Of these, most (83.6%; N = 215) used OTC medication at least once per week; 31.1% (N = 80) used it daily, 27.6% (N = 71) used it four to six times per week, 24.9% (N = 64) used it one to three times per week. Oral antihistamines were the most commonly used OTC medication (59.1%; N = 152); decongestants, corticosteroids nasal and oral sprays/drops and eye drops were each used by 52.1% (N = 134), 47.5% (N = 122) and 27.6% (N = 71) subjects, respectively.

Of all subjects with perennial allergies (N = 511), 79.6% (N = 407) had received treatment prescribed by a healthcare professional (HCP) for their allergy either prior to or at the time of the survey. Forty-four percent (N = 225) of all subjects had ever received AIT, which was similar across the four markets (31.6–52.0%). At the time of the survey, 20.5% (N = 105) of all subjects were receiving AIT.

### Frequency of sleep problems

**Fig. 2:**
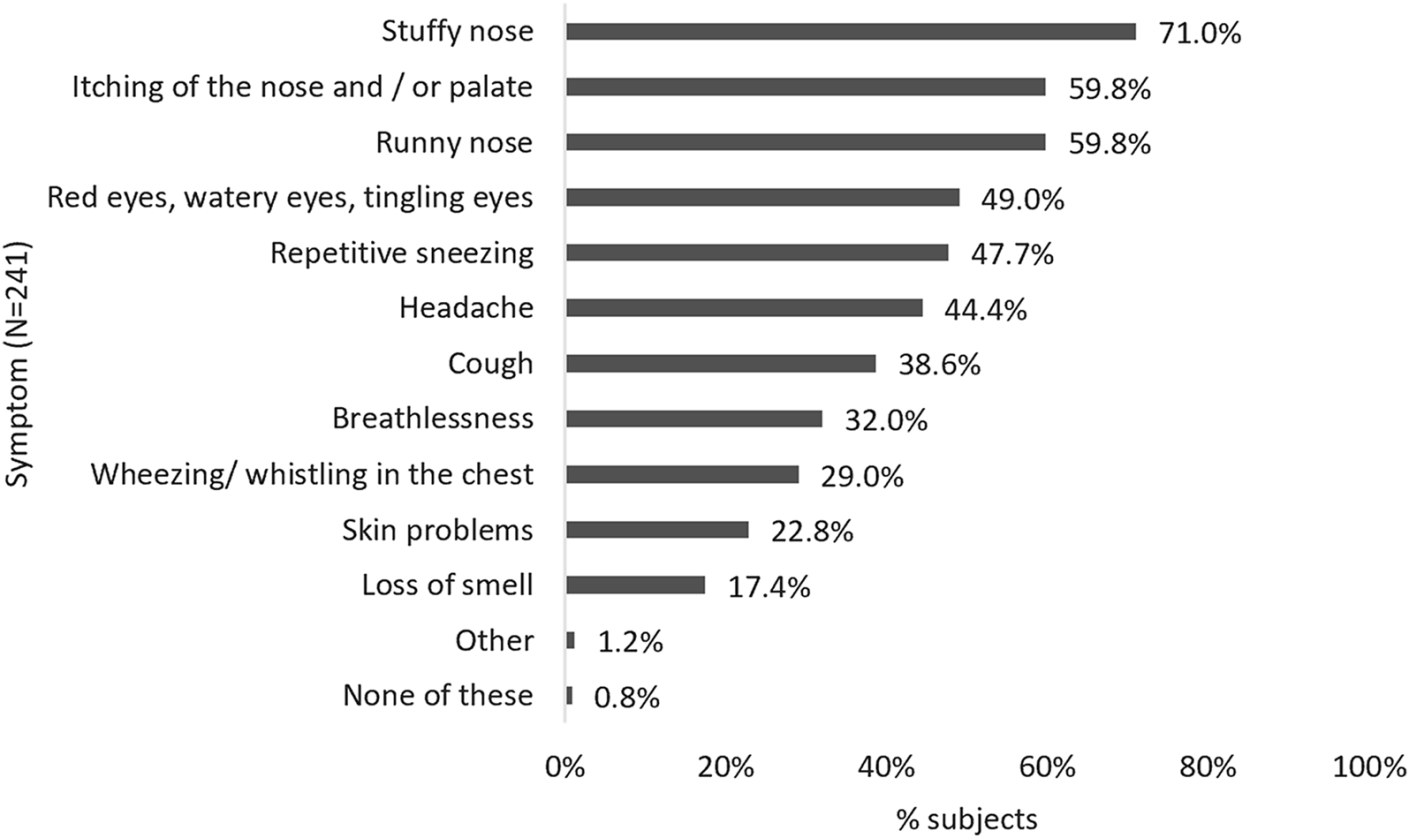
Symptoms affecting sleep in all subjects reporting AR-related sleep problems

Of all subjects with perennial allergies, 66.1% reported sleep issues (N = 338), and 47.1% reported sleep issues that were related to their AR (N = 241). Nasal symptoms such as a stuffy/runny nose or itching of the nose/palate and other head-symptoms were most commonly reported to affect sleep (Fig. 2), yet 55 (10.1%) attributed their sleep issues to skin problems.

**Fig. 3:**
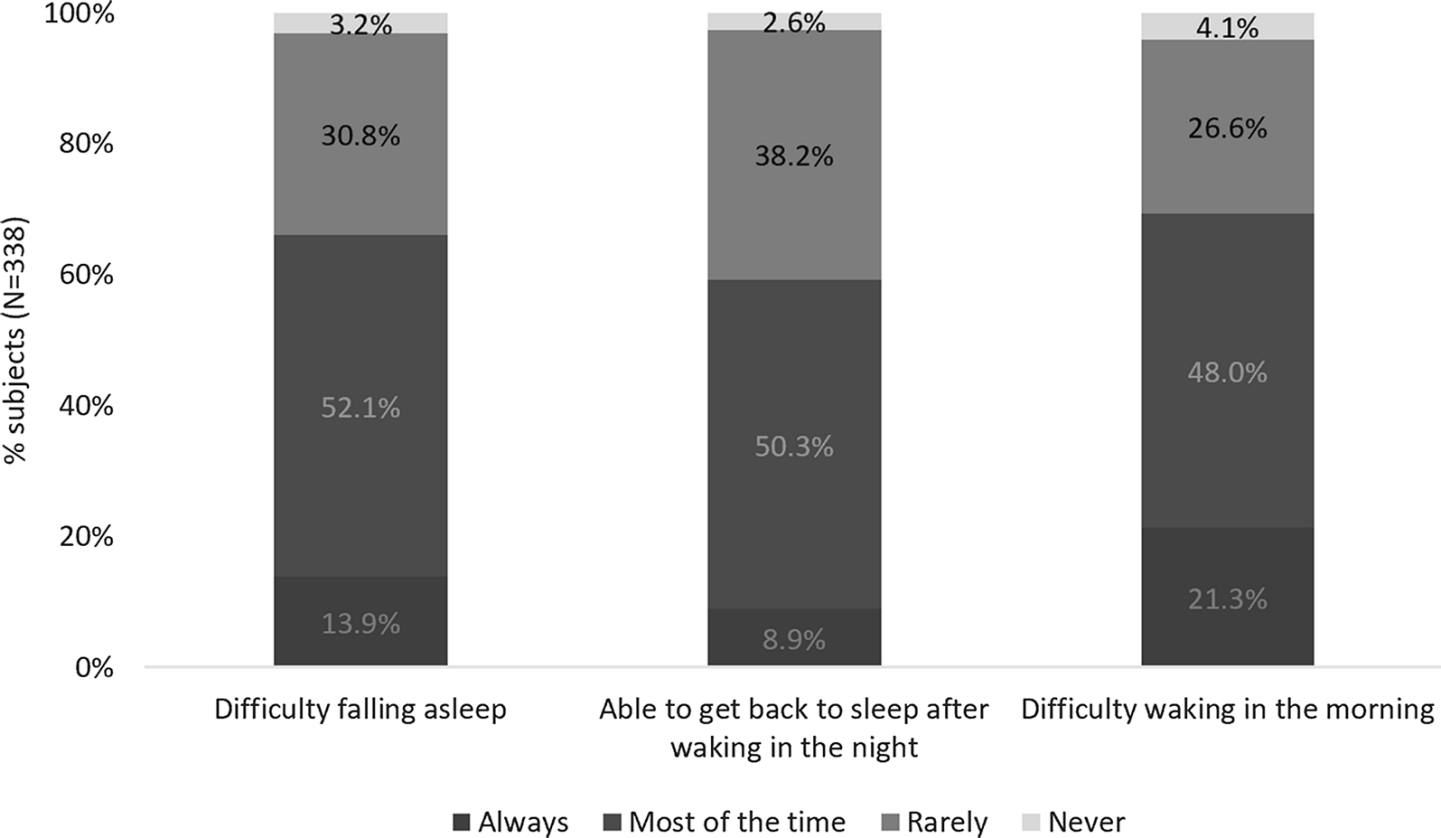
Frequency of sleep problems in all subjects with reported sleep problems

On average, subjects with sleep issues woke 3.8 times per night, with 92.0% (N = 311) taking over 15 min to fall asleep and 22.2% (N = 75) over 60 min. Sixty-six percent (N = 223) had difficulties falling asleep at least most of the time and 40.8% (N = 138) reported that they were rarely or never able to get back to sleep after waking in the night. The majority (69.2%; N = 234) also had difficulties waking in the morning most or all of the time (Fig. 3). These results were similar in the population who reported their sleep issues to be related to AR (N = 241) (Additional file 3).

Snoring was reported in 76.6% (N = 259) of the population with sleep problems; 26.3% (N = 89) snored always and 50.3% (N = 170) sometimes. The majority of those who snored (76.8%; N = 199) had been told they snored loudly and just under half (46.7%; N = 121) of those who snored reported being told that they stopped breathing while sleeping—potentially indicative of sleep apnoea.

### Impact of sleep problems

**Fig. 4:**
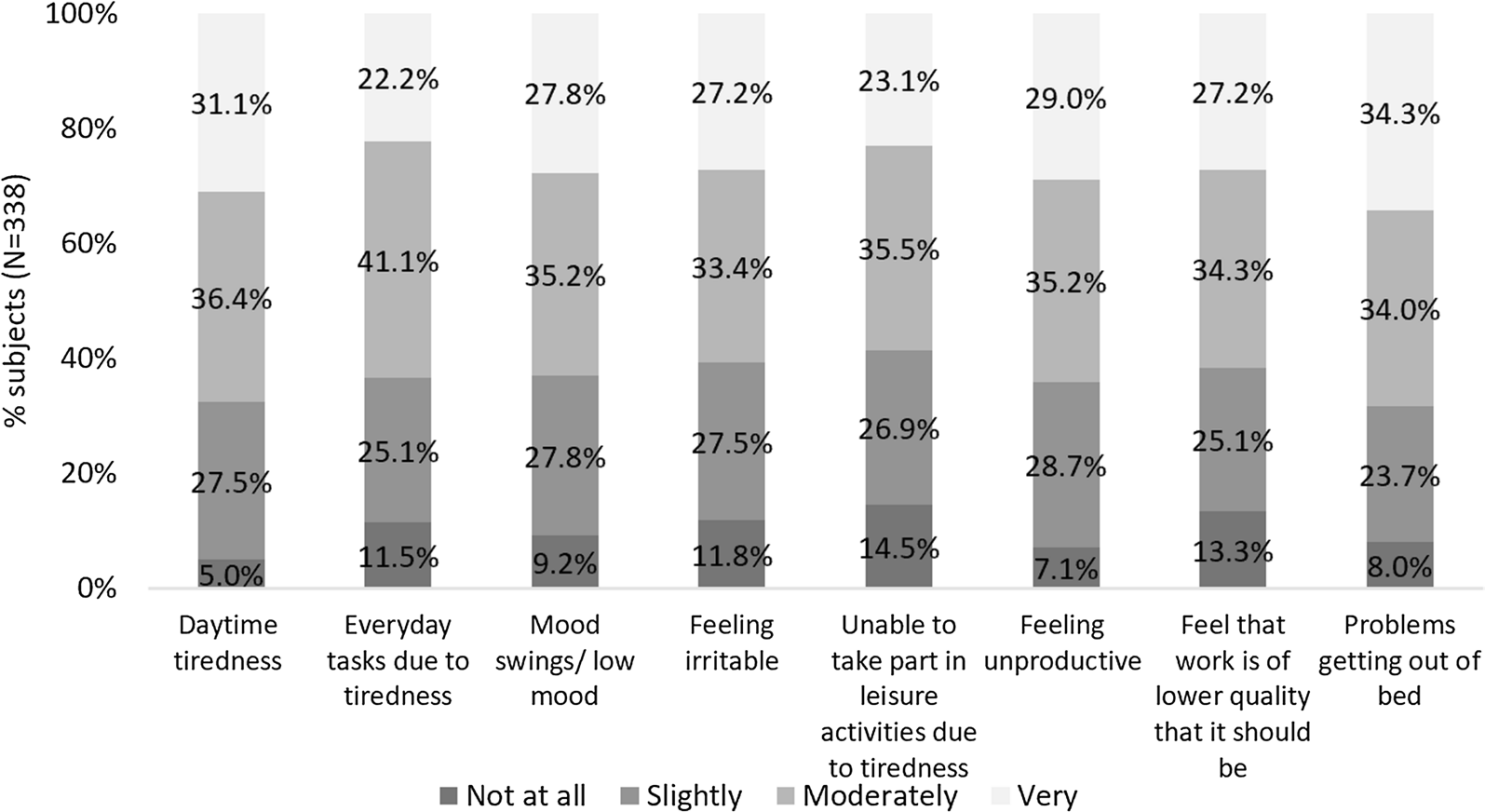
Impact of sleep in all subjects who reported sleep problems

Disturbances in daily functioning due to the impact of AR on sleep were reported in 85.5–95.0% (N = 289–321) of subjects with sleep problems across all aspects investigated (Fig. 4).

Sleep problems caused concern in almost all subjects with sleep problems (95.6%; N = 323). A total of 55.9% (N = 189) were unsatisfied with their sleep and 24.3% (N = 82) were very unsatisfied.

### Impact on productivity according to the WPAI

WPAI scores indicated that sleep impacted on work, productivity and activity in perennial allergy sufferers. In the full sample of subjects (N = 511), 63.6% (N = 325) were employed and included in the WPAI analysis. The average work time missed (absenteeism) over the past 7 days was 12.0%, impairment while working due to health (presenteeism) was 46.9%, overall work impairment due to health (absenteeism plus presenteeism) was 53.3% and activity impairment due to health (non-work related) was 47.1%.

### Sub-analysis: sleep problems and impact in those with AR and AA compared to AR alone

**Fig. 5:**
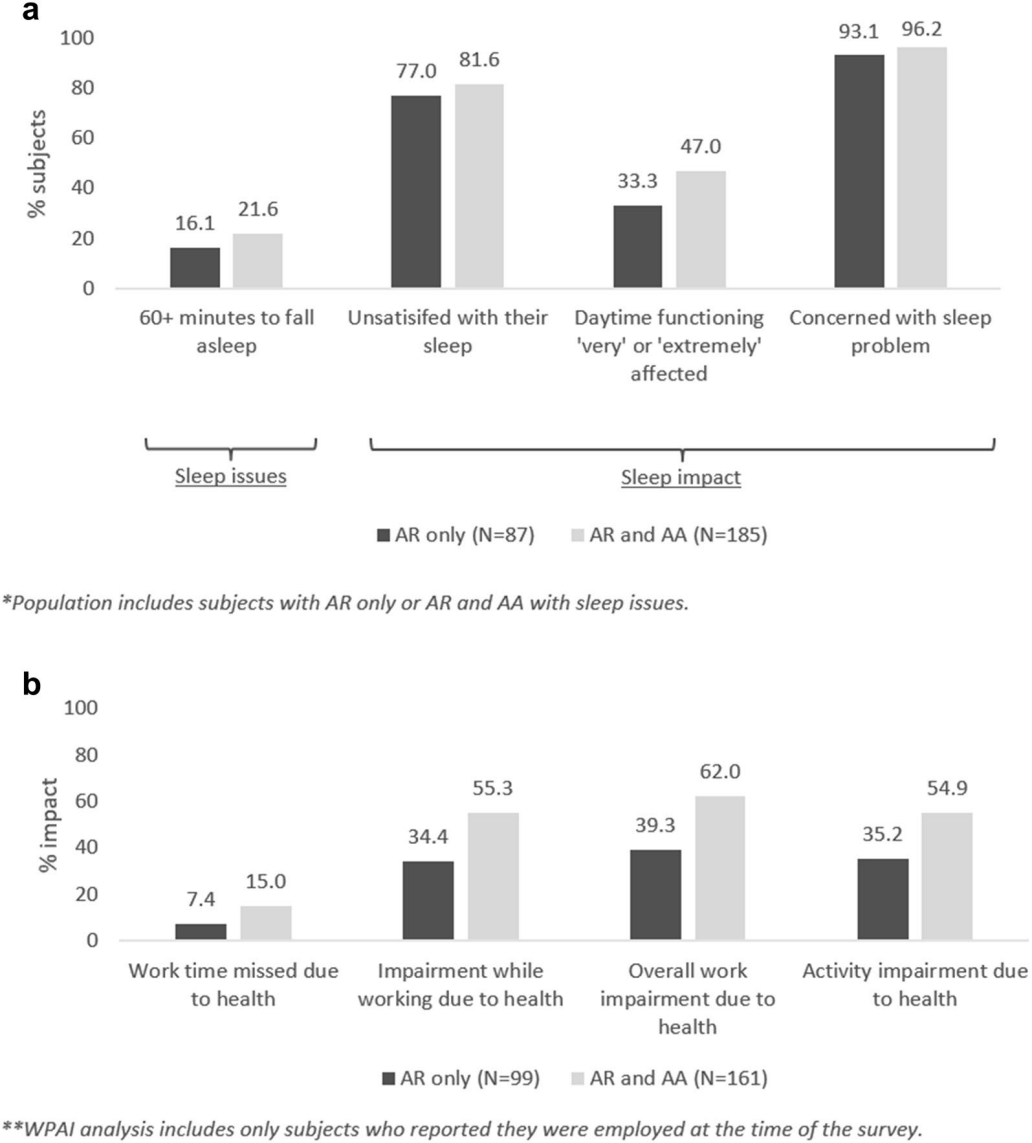
**a** Sleep problems and **b** WPAI total scores in subjects with physician diagnosed AR only versus AR and AA

This sub-analysis considered those with a physician diagnosis of both AR and AA [46.3% of all subjects with perennial allergy (N = 237)], versus those diagnosed with AR alone [31.1% (N = 159)]. In those with diagnosed AR and AA, sleep problems were more frequent than in AR alone [78.1% (N = 185) versus 54.7% (N = 86)] and more burdensome (Fig. 5). For instance, those with both AA and AR diagnoses woke an additional time per night (3.9 versus 3.0), and a higher proportion took over 60 min to fall asleep, were unsatisfied or concerned with their sleep and reported daytime functioning to be ‘very’ or ‘extremely’ affected due to sleep (Fig. 5).

Work and activity were also impaired to a greater degree in those with both AR and AA versus AR alone. Those diagnosed with AR and AA saw an overall impairment of 62.0% and 54.9% for work and activity respectively, versus 39.3% and 35.2% for those with AR diagnosis only (Fig. 5).

### Sub-analysis: characteristics of and sleep problems in those treated with AIT versus other prescribed treatment

Characteristics of those who were receiving AIT at the time of the survey [20.5% (N = 105)] were compared to those who were receiving any other prescription treatment but not AIT [21.5% (N = 110)]. The average age of subjects on AIT was lower than those on non-AIT prescription medication (33.9 years versus 42.6 years), but gender, smoking status, proportion of those with perennial only allergies and polysensitisation to allergens was similar between the groups. Having a diagnosis of AA in addition to AR was more common in those on AIT than on other prescribed treatments [68.6% (N = 72) versus 58.2% (N = 64)], as was persistent disease as per ARIA criteria [87% (N = 87) versus 60% (N = 66)] and OTC medication use [72.4% (N = 76) versus 42.7% (N = 47)].

**Fig. 6:**
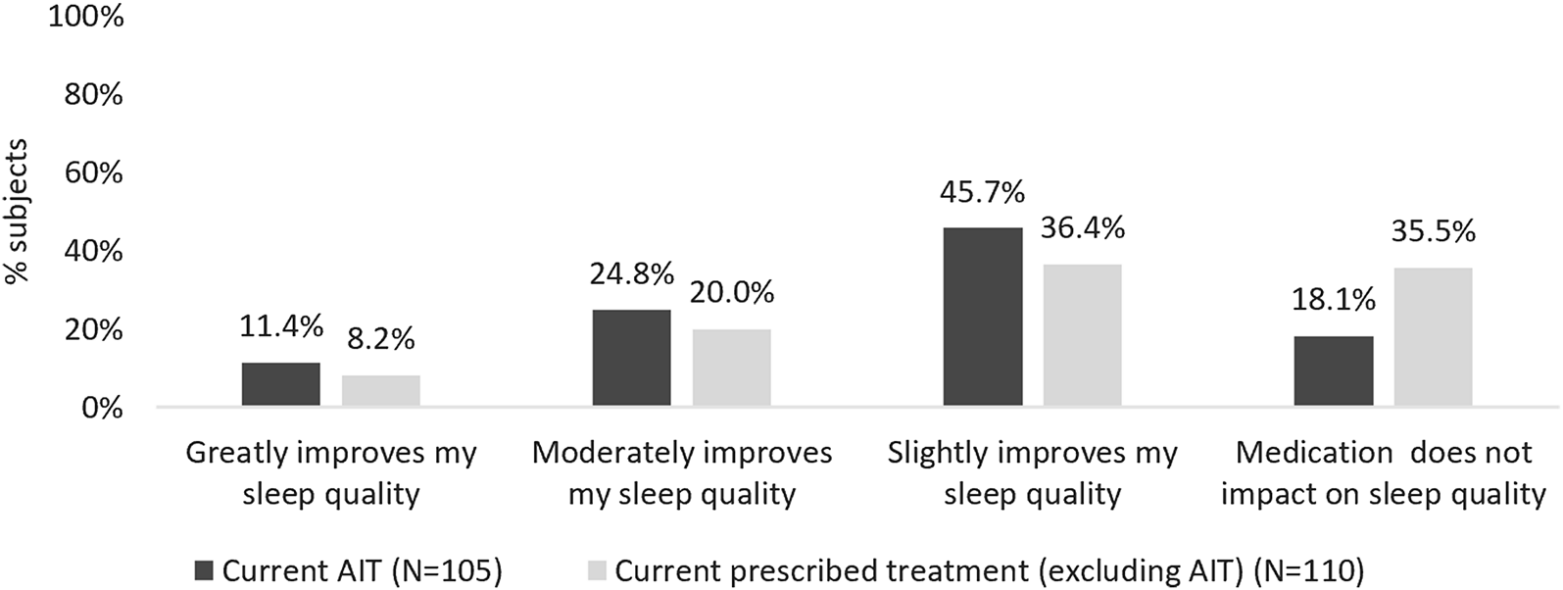
Sleep improvements reported in all subjects receiving AIT versus other prescription medication

Subjects receiving AIT more frequently reported moderate [24.8% (N = 26)] or great [11.4% (N = 12)] improvement in sleep due to allergy treatment than those on other prescribed treatments (Fig. 6). Additionally, reported levels of satisfaction with sleep in those with sleep problems were higher in the AIT-group [28.2% (N = 24) versus 15.7% (N = 11)].

## Discussion

This study identified that in those with perennial AR, sleep problems are very common and disruptive to everyday life. This aligns to previous research, which demonstrated that there is a high sleep-related burden associated with AR in general and with perennial AR in particular [11, 13, 20]. However, this study further established that the prevalence and associated burden in those with diagnoses of both perennial AR and AA is greater than in AR alone. It is clear that perennial allergy sufferers experience symptoms beyond classical sneezing, rhinorrhoea and nasal congestion, including great impact on sleep, subsequent functioning, and quality of life.

Results indicate that work and activity performance is greatly impacted by perennial AR. The WPAI analysis indicated as much as 12.0% of worktime was missed (absenteeism) due to sleep problems. Specifically, this equates to 4.8 h a week per subject working the average European week of 40.3 h [22]. Further, the degree to which productivity at work was impaired at 46.9% (presenteeism) indicates a substantial disruption in work due to sleep issues. Addressing sleep issues associated with perennial allergy is therefore important from the societal perspective as well as the patient perspective and should be considered in treatment management.

Our results are aligned with a previous cross-sectional French study, which characterised the impact of sleep disorders within a population of HDM allergy sufferers initiating AIT treatment (N = 1750)—the MORPHEE study [11]. The prevalence of sleep issues in the MORPHEE study compared to the current study (73.5% versus 66.1%) were broadly similar considering differences in sampling and the current study being multi-country. The slightly higher prevalence reported in the MORPHEE study may be due to the population investigated: the MORPHEE study captured data from subjects as they were initiating AIT, which is indicated for those with severe and/or persistent AR that is difficult to control with symptomatic treatments alone, thereby capturing a more severe population than the present analysis of which only 20.5% were receiving AIT at the time of the study [23]. Nevertheless, it should be noted that both estimates of sleep problems within the perennial AR population were more than double that of general population estimates in Western Europe from 2008 [24]. Further, the prevalence of night time awakenings in our study was far beyond that in a US population study [25]. It must be noted, however, that there has been limited recent research in the general population which investigates the specificities of sleep problems. Therefore, although our results indicate that sleep issues are apparent in the perennial allergy population, further studies with control-group comparison are required to confirm this.

Given the number of subjects identified receiving AIT at the time of the survey, analyses into the characteristics and treatment satisfaction/improvement were conducted in this group. Characteristics indicated that AIT had been prescribed to a population with more severe disease as those on AIT more often reported persistent disease and diagnosis of both AA and AR than those not on AIT. However, it should not be overlooked that the more severe population may have been more likely to answer the survey. Those receiving AIT reported greater improvements in sleep due to medication than those on other prescription medications, which may indicate a positive impact of AIT on sleep—however, as this study was not designed to investigate AIT effectiveness, further controlled research is required to corroborate this. Improvements in sleep associated with AIT have previously been reported in perennial AR by Novakova et al. [19] in a real-world study; significant improvements in the sleep-specific dimension of the Rhinoconjunctivitis Quality of Life Questionnaire (RQLQ) were observed following a 3-year AIT course compared to baseline. Further, recent clinical trial analysis by Jacobi et al. [26] indicates a significant improvement in sleep, based on RQLQ, following treatment with HDM sublingual immunotherapy (SLIT) tablets.

AIT has also demonstrated effects in AA, whereby a phase III, multicentre European RCT study (N = 834), demonstrated improvements in moderate-severe asthma exacerbations following SLIT-tablet treatment for HDM allergy [27]. Of note, a greater burden of disease when AA was present was indicated in the results of the current study, including increased frequency and severity of sleep issues as well as increased work and activity impairment. The higher burden of disease associated with AA diagnosis should therefore be considered in treatment decisions.

Further research is required, including real-world data collection, to confirm the potential for AIT in those with perennial AR-related sleep issues as well as those with associated AA. Indeed, the ongoing CARIOCA trial (NCT03746860) is investigating AIT efficacy (HDM SLIT-tablet) and impact on sleep in those with AR and AA in clinical practice [28].

### Limitations

The present study was designed to capture those with perennial AR—however, some characteristics of the subjects identified merit discussion. Firstly, the majority (> 90%) had moderate-to-severe disease according to ARIA classification, which was unexpected as the proportion with moderate-to-severe disease is much lower in the general population, reported at around 67% [11]. This may be due to those with mild AR not identifying themselves as having a respiratory allergy, and therefore not self-reporting and being included in the survey. Therefore, characteristics specific to the moderate-severe population were overrepresented and the prevalence of sleep issues (66.1%) should not be generalised to the mild AR population. Secondly, a large proportion of the subjects were receiving AIT at the time of the survey (20.5%) or had been prescribed AIT at some point in time (44.0%). Potential explanations for this high incidence of AIT treatment include the high proportion of moderate-to-severe subjects identified, indicating a greater proportion may be eligible for AIT than in the general perennial allergy population. Finally, a small number of subjects who reported AR-related sleep problems (N = 6) reported no effect on sleep within the last month. Although unexpected, it may be because the recall period was 1 month, and the study was conducted in the Autumn/Winter period, in which sleep issues were reported as being least prevalent, in particular for those with additional seasonal allergies. Therefore, some subjects may experience sleep problems in general but had not experienced them within the recall period of the survey.

The sampling undertaken was geographically representative but did not use quotas to explicitly reflect demographics identified in previous AR assessments, limiting generalisability. Further, study quotas of 100 per country were met in France and Germany but were slightly under in Denmark (N = 89) and Sweden (N = 98) within the agreed survey period. This is likely due to the mild population not being identified and the Nordic country populations being significantly smaller than France and Germany, resulting in a smaller pool of potential respondents. The consequent overrepresentation of France and Germany in the results may have led to some country-specific bias within the data. However, as demographic data such as age, sex, smoking status and severity of disease did not differ widely between countries, the impact is considered likely to be minimal.

The duration of treatment was not measured and therefore treatment effects could not be analysed. Therefore, it is not possible to say whether there is a causal link between the apparent impact on sleep quality seen when those on AIT were compared to those on other prescription treatments. Further, the study was not designed to capture confounding factors—for example, methods that patients used to alleviate their exposure to allergens, such as closing windows and washing sheets, which would be interesting to investigate in future research.

Finally, limitations inherent to the study design were apparent: the survey relied on subjects self-reporting, and so some inaccuracy may have been introduced either based on subject recall or errors in completing the survey (i.e. contradictory results)—for example, more subjects self-reported pollen allergy than reported having seasonal allergies.

## Conclusions

In perennial AR sufferers, sleep problems are prevalent and impact on daily functioning, including disturbance of work and completion of everyday task and leisure activities. Results are indicative of a greater burden of sleep problems in those with diagnoses of both AR and AA compared to AR alone. However, given the exploratory nature of this survey, continuing controlled investigation is essential in order to deduce the precise relationship between perennial allergy, sleep and the impact of allergy treatment.

## Supplementary information

**Additional file 1.** Questionnaire.

**Additional file 2.** Routing of questions in the sleep section (A) and treatment (B) section of the survey.

**Additional file 3.** Frequency of sleep problems in all subjects who reported their sleep problems were due to their AR.

## Data Availability

The datasets used and/or analysed during the current study are available from the corresponding author, MRR, on reasonable request.

## Abbreviations

AIT: allergen immunotherapy
AA: allergic asthma
AR: allergic rhinitis
ARIA: allergic rhinitis and its impact on asthma
HDM: house dust mite
HCP: health care practitioner
OTC: over the counter
RQLQ: Rhinoconjunctivitis Quality of Life Questionnaire
SLIT: sublingual immunotherapy
WHO: World Health Organisation
WPAI: Work Productivity and Activity Index
