# Correction to: The impact of perennial allergic rhinitis with/without allergic asthma on sleep, work and activity level

**DOI:** 10.1186/s13223-020-0408-4

**Published:** 2020-02-05

**Authors:** Mercedes Romano, Stephanie James, Emily Farrington, Richard Perry, Lisa Elliott

**Affiliations:** 1 ALK, Bøge Alle 1, 2970 Hørsholm, Denmark; 2 Adelphi Values, Macclesfield, UK

## Correction to: Allergy Asthma Clin Immunol (2019) 15:81 10.1186/s13223-019-0391-9

The original version of this article [1] unfortunately included an error to an author’s name and citation.

The first and corresponding author was unfortunately presented as Mercedes Rodriguez Romano. The correct author name is Mercedes Romano.

The correct author name has been included in the author list of this Correction article.
