# Supplementary material for: The impact of perennial allergic rhinitis with/without allergic asthma on sleep, work and activity level
Source: Allergy Asthma Clin Immunol. 2019 Dec 6;15:81. doi: 10.1186/s13223-019-0391-9 (PMC6896721; doi:10.1186/s13223-019-0391-9)
Supplement: Supplementary file 1 — Additional file 1. Questionnaire. [file 13223_2019_391_MOESM1_ESM.docx]

1. **In which season(s) are your allergy symptoms worst? (several answers possible)**

| Spring |  | □ | |
| --- | --- | --- | --- |
| Summer |  | □ |  |
| Autumn |  | □ |  |
| Winter |  | □ |  |
| The same all year | | □ |  |
|  |  |  |  |

1. **When you are suffering from allergic rhinitis:**
   1. **Do the symptoms normally last more than four days in a week?**

| Yes | No |
| --- | --- |
| □ | □ |

- 1. **Do you experience symptoms for more than four weeks in a row?**

| Yes | No |
| --- | --- |
| □ | □ |

1. **How do your symptoms affect you?**

*Answer YES for any of the statements listed below that apply. Answer NO for those that do not*.

My symptoms disturb my sleep

| Yes | No |
| --- | --- |
| □ | □ |

My symptoms restrict my daily activities (such as self-care, eating, mobility, working, driving, interacting with others or leisure time.)

| Yes | No |
| --- | --- |
| □ | □ |

My symptoms restrict my participation in school or work

| Yes | No |
| --- | --- |
| □ | □ |

My symptoms are troublesome to me

| Yes | No |
| --- | --- |
| □ | □ |

**Sleep symptoms**

1. **Do you have sleep problems related to your allergy?**

| Yes | No |
| --- | --- |
| □ | □ |

**If NO, go to section 2.4**

1. **In which season(s) are your sleep problems worst? (several answers possible)**

| Spring |  | □ | |
| --- | --- | --- | --- |
| Summer |  | □ |  |
| Autumn |  | □ |  |
| Winter |  | □ |  |
|  | **or** |  |  |
| The same all year |  | □ |  |
|  |  |  |  |

1. **How often do your allergy symptoms affect your sleep (thinking of the last month)?**

| Never | Rarely | Most of the time | Always |
| --- | --- | --- | --- |
| □ | □ | □ | □ |
|  |  |  |  |

**If Never, go to section 2.4**

1. **Are you satisfied with your current sleep (thinking of the last month)?**

| Very unsatisfied | Unsatisfied | Satisfied | Very satisfied |
| --- | --- | --- | --- |
| □ | □ | □ | □ |

1. **Do you have difficulty falling asleep (thinking of the last month)?**

| Never | Rarely | Most of the time | Always |
| --- | --- | --- | --- |
| □ | □ | □ | □ |

1. **How many minutes does it usually take for you to fall asleep (thinking of the last month)?**

| Less than 15 mins | 15 to 30 mins | Over 30 and up to 60 mins | More than 60 mins |
| --- | --- | --- | --- |
| □ | □ | □ | □ |

1. **How many times per night do you usually wake up (thinking of the last month)?**

________ (write in number) Don’t know □

1. **How many times per night do you wake up due to your allergy symptoms?**

________ (please state) Don’t know □

1. **When you wake up in the night, can you fall back to sleep (thinking of the last month)?**

| Never | Rarely | Most of the time | Always |
| --- | --- | --- | --- |
| □ | □ | □ | □ |

1. **Do you experience difficulties waking up in the morning due to tiredness (thinking of the last month)?**

| Never | Rarely | Most of the time | Always |
| --- | --- | --- | --- |
| □ | □ | □ | □ |

1. **Do you ever snore?**

| Never | Sometimes | Always | Don’t know |
| --- | --- | --- | --- |
| □ | □ | □ | □ |

1. **Have you ever been told that you snore loudly?**

| Yes | No |
| --- | --- |
| □ | □ |

1. **Have you ever been told that you stopped breathing for several seconds whilst you slept?**

| Yes | No |
| --- | --- |
| □ | □ |

1. **Which allergy symptoms cause difficulty with falling asleep/waking up?**

| Red eyes, watery eyes, tingling eyes | □ | Headache | □ |
| --- | --- | --- | --- |
| Runny nose | □ | Wheezing/ whistling in the chest | □ |
| Stuffy nose | □ | Breathlessness | □ |
| Repetitive sneezing | □ | Cough | □ |
| Itching of the nose and / or palate | □ | Skin problems | □ |
| Loss of smell | □ |  |  |

**Impact of sleep symptoms**

1. **To what extent do your sleep difficulties disturb your daily functioning?**

*Daily functioning is defined by ability to complete tasks of everyday life, these include: self-care, eating, mobility, working, driving, interacting with others or leisure time.*

| None | Slightly | Very | Extremely |
| --- | --- | --- | --- |
| □ | □ | □ | □ |

1. **To what extent are you concerned about your sleep problems?**

| None | Slightly | Moderately | Very | Extremely |
| --- | --- | --- | --- | --- |
| □ | □ | □ | □ | □ |

1. **To what extent do you struggle with the following due to tiredness or interrupted sleep (thinking of the last month):**

| **Daytime tiredness** | | |  |  |
| --- | --- | --- | --- | --- |
| None | Slightly | | Moderately | Very |
| □ | □ | | □ | □ |
| **Everyday tasks due to tiredness** | | |  |  |
| None | Slightly | | Moderately | Very |
| □ | □ | | □ | □ |
| **Mood swings/ low mood** | | |  |  |
| None | Slightly | | Moderately | Very |
| □ | □ | | □ | □ |
| **Feeling irritable** | | |  |  |
| None | Slightly | | Moderately | Very |
| □ | □ | | □ | □ |
| **Unable to take part in leisure activities due to tiredness** | | | | |
| None | Slightly | | Moderately | Very |
| □ | □ | | □ | □ |
| **Feeling unproductive** | | |  |  |
| None | Slightly | | Moderately | Very |
| □ | □ | | □ | □ |
| **Feel that work is of lower quality that it should be** | | | | |
| None | Slightly | | Moderately | Very |
| □ | □ | | □ | □ |
| **Problems getting out of bed** | |  |  |  |
| None | Slightly | | Moderately | Very |
| □ | □ | | □ | □ |

1. **How satisfied are (were) you with your most recent medication(s) from a health care professional to relieve your allergy symptoms?**

| Very unsatisfied | Unsatisfied | Satisfied | Very satisfied |
| --- | --- | --- | --- |
| □ | □ | □ | □ |

1. **How do you think your treatment for allergy has improved your quality of sleep?**

| Medication does not impact on sleep quality | Not at all | Slightly | Very |
| --- | --- | --- | --- |
| □ | □ | □ | □ |

1. **Do you buy any medications to help you sleep which do not require a prescription?**

| Yes | No |
| --- | --- |
| □ | □ |

1. **How often do you take this/these medications? (thinking of the last month)**

| Every day | 4-6 times per week | 1-3 times per week | Less than once per week |
| --- | --- | --- | --- |
| □ | □ | □ | □ |

**Impact on work and daily life**

Note: the WPAI-GH was adapted to include sleep problems as the health problem.

1. Are you currently employed (working for pay)?

**YES/NO,**  If **NO**, skip to question 30

The next questions are about the **past seven days**, not including today.

1. **During the past seven days, how many hours did you miss from work because of problems associated with sleep problem/tiredness?**

*Include hours you missed on sick days, times you went in late, left early, etc., because of your* sleep problem/tiredness*. Do not include time you missed to participate in this study.

_____* HOURS

1. **During the past seven days, how many hours did you miss from work because of any other reason, such as vacation, holidays, time off to participate in this study?**

   _____HOURS
2. **During the past seven days, how many hours did you actually work?**

   _____hours *(If “0”, skip to question 30)*
3. **During the past seven days, how much did sleep problems/tiredness affect your productivity while you were working?**

*Think about days you were limited in the amount or kind of work you could do, days you accomplished less than you would like, or days you could not do your work as carefully as usual. If sleep problem/tiredness affected your work only a little, choose a low number. Choose a high number if sleep problem/tiredness affected your work a great deal.*

Consider only how much sleep problems/tiredness affected your
productivity while you were working (circle a number)

| sleep problems/ tiredness had no effect on my work |  |  |  |  |  |  |  |  |  |  |  | sleep problems/ tiredness completely prevented me from working |
| --- | --- | --- | --- | --- | --- | --- | --- | --- | --- | --- | --- | --- |
|  | 0 | 1 | 2 | 3 | 4 | 5 | 6 | 7 | 8 | 9 | 10 |  |

1. **During the past seven days, how much did your sleep problems/tiredness affect your ability to do your regular daily activities, other than work at a job?**

   *By regular activities, we mean the usual activities you do, such as work around the house, shopping, childcare, exercising, studying, etc. Think about times you were limited in the amount or kind of activities you could do and times you accomplished less than you would like. If* sleep problem/tiredness *affected your activities only a little, choose a low number. Choose a high number if* sleep problems/tiredness *affected your activities a great deal.*

Consider only how much sleep problems/tiredness affected your ability
to do your regular daily activities, other than work at a job (circle a number)

| sleep problems/ tiredness had no effect on my daily activities |  |  |  |  |  |  |  |  |  |  |  | sleep problems/ tiredness completely prevented me from doing my daily activities |
| --- | --- | --- | --- | --- | --- | --- | --- | --- | --- | --- | --- | --- |
|  | 0 | 1 | 2 | 3 | 4 | 5 | 6 | 7 | 8 | 9 | 10 |  |

**Thank you for your time**
