# Supplementary figures and images for: The impact of perennial allergic rhinitis with/without allergic asthma on sleep, work and activity level
Source: Allergy Asthma Clin Immunol. 2019 Dec 6;15:81. doi: 10.1186/s13223-019-0391-9 (PMC6896721; doi:10.1186/s13223-019-0391-9)

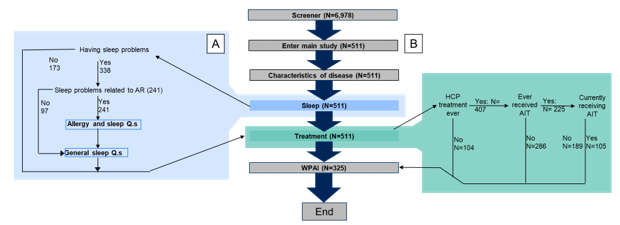

Supplement: Supplementary file 2 — Additional file 2. Routing of questions in the sleep section (A) and treatment (B) section of the survey. [file 13223_2019_391_MOESM2_ESM.png]

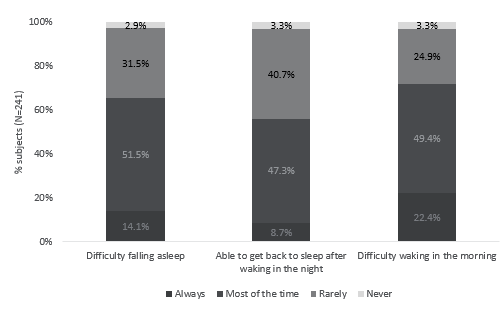

Supplement: Supplementary file 3 — Additional file 3. Frequency of sleep problems in all subjects who reported their sleep problems were due to their AR. [file 13223_2019_391_MOESM3_ESM.png]
